# The “Bergamo Approach” for Pediatric and Adolescent Polytrauma—A One-Center Experience

**DOI:** 10.3390/children12091194

**Published:** 2025-09-08

**Authors:** Nicola Guindani, Maurizio Cheli, Daniela Ferrari, Giovanna Colombo, Ezio Bonanomi, Federico Chiodini, Maurizio De Pellegrin

**Affiliations:** 1 Orthopedics and Traumatology Unit, Regional Health Care and Social Agency Papa Giovanni XXIII, Regional Hub for the Pediatric Polytrauma, 24127 Bergamo, Lombardia, Italy; 2 Pediatric Surgery Unit, Regional Health Care and Social Agency Papa Giovanni XXIII, Regional Hub for the Pediatric Polytrauma, 24127 Bergamo, Lombardia, Italy; 3 Pediatric Intensive Care Unit, Regional Health Care and Social Agency Papa Giovanni XXIII, Regional Hub for the Pediatric Polytrauma, 24127 Bergamo, Lombardia, Italy

**Keywords:** pediatric polytrauma, pediatric major trauma, damage control in pediatric polytrauma

## Abstract

**Introduction.** Pediatric polytrauma (PPT) and major trauma in pediatric patients (PMT) present unique challenges compared to adult trauma care due to distinct anatomical and physiological differences. PPT/PMT remains the leading cause of death in children, responsible for over 50% of pediatric deaths and 15% of pediatric hospital admissions due to its long-term effects. This single-institution study focuses on the initial management of PPT/PMT from an orthopedics and traumatology point of view. **Material and Methods.** In the present study, data of PPT/PMT managed in one single institution, an academic level I pediatric trauma center, in patients <18 years of age, were analyzed over different periods. Over a 10-year period, diaphyseal femur fractures were analyzed as indicators of damage control (DCO) versus definitive treatment. Over a 4-year period (2021–2024), the associated lesions of PPT (head injuries, thoracic and abdominal lesions, spine lesions, major blood vessel lesions, and major musculoskeletal injury) were analyzed. Over a 1-year period (2019), the overall in-hospital mortality and admission rates in the pediatric intensive care unit were analyzed. **Results.** In the 10-year period, among 298 diaphyseal femur fractures, 46/298 (15%) were classified as PPT in which DCO was performed according to age as follows: in the age-group 15–17 years 23/23 (100%) with temporary external fixation (ExFix); in the age group 12–14 years, 9/14 (64%) with ExFix and 5/14 (26%) and elastic stable intramedullary nails (ESINs); in the age group 5–11 years, 1/5 (20%) with ExFix and 4 with ESIN; in the age group 0–4 years, 2/4 (50%) with ESIN and 2/4 (50%) with a cast. In the 4-year period, PPT/PMTs were associated with 60% head injury, 25% thoracic lesion(s), 18% abdominal lesion(s), 16% spine injury, 5% lesion of a major blood vessel, and 30% major musculoskeletal injuries. In 2019, there were 193 patients admitted to the emergency room as PPT/PMT: 115 were ≤12 years old and 78 were >12 years old. On admission, 46% were admitted to the pediatric intensive care unit, and 65% were admitted to the department of traumatology as inpatients. The in-hospital mortality rate was 7%. **Discussion and Conclusions.** In our institution, pediatric trauma is assessed using the Pediatric Trauma Score (PTS), and the workup follows the ATLS guidelines with a dedicated trauma team. The role of the orthopedic surgeon during the primary evaluation of PPT/PMT is to contribute to stopping bleeding and hemorrhagic shock. In PPT/PMT, DCO in adolescents is superimposable to adults, whilst in babies and children, DCO is still performed, but it is not a form of temporary external fixation.

## 1. Introduction

Pediatric polytrauma (PPT) and major trauma in pediatric patients (PMT) present unique challenges compared to adult trauma care due to distinct anatomical and physiological differences. PPT/PMT remains the leading cause of death in children; it is responsible for over 50% of pediatric deaths [1,2,3] and 15% of pediatric hospital admissions due to the long-term effects. In the EU, the leading causes of trauma are road traffic accidents; in the US, on the contrary, up to 50% of the deaths are caused by firearms [3,4,5], whilst the rate of suicide attempts is ubiquitously increasing at a worrying pace [6].

The rate of non-accidental trauma in PPT/PMT is inversely proportional to age and can be as high as 32% in infants [7]; therefore, the anamnesis and background of the trauma must be cleared, at least before discharge [4,8,9,10,11,12]. A diaphyseal femur fracture in non-walking age children is considered “the smoking gun” of NAT [4,7,13]. Considering both the acute management and the following consequences, the social burden of PPT/PMT is enormous [3,14,15,16,17]. The main causes of mortality in PPT/PMT are intracranial lesions (75%), followed by thoracic trauma (25%) and abdominal injuries (10%) [18,19,20]. The most common causes of long-term functional deficits in PPT/PMT are due to injuries to the central nervous and musculoskeletal systems [21,22]; in other words, musculoskeletal injuries in PPT/PMT are very common and mostly affect morbidity, whilst mortality is mostly linked to head and trunk injuries [19,22,23]. The epidemiology of fractures in PPT/PMT differs from that of isolated fractures in children and polytrauma in adults. In PPT/PMT, most fractures involve long bones of the lower extremities, followed by the pelvis and upper extremities. In comparison to adults, fractures (particularly pelvic fractures) occur less frequently [19,24,25] and parenchymatous lesions are less likely to be associated with fractures. This is particularly relevant for the pelvis and thorax [4,19,23], where inconspicuous X-ray findings could lead to an underestimation of the underlying organ lesions. Up to 50–60% of PPT cases have orthopedic lesions, with long bone fractures being more frequent: in order of frequency, tibia and femur for the lower limb and humerus and radius for the upper limbs, respectively. In children, 25% of all diaphyseal femur fractures are associated with other lesions, and 16% occur in PPT [7,26,27]. For those reasons, the association between PPT/PMT and diaphyseal femur fracture in children is extensively studied [12,23,28].

Major trauma is a time-dependent disease, and management starts with pre-hospital rescue services and the meticulous organization of the trauma network [5,29]. Specialized pediatric trauma centers best comply with the need for specialized care in handling PPT/PMT: treatment in centers with pediatric intensive care units (PICUs) demonstrates better results in comparison with general (adult) intensive care units, emphasizing the importance of targeted pediatric care [30,31]. This is worthwhile both for children and adolescents and accounts for lower mortality rates, better long-term results [32], and a more conservative approach in comparison to adult trauma centers [30,32,33,34,35].

The care of PPT/PMT requires specialized knowledge of age-related anatomical and physiological characteristics, and a tailored approach according to age is the cornerstone of treatment [36,37]. Key challenges in pediatric trauma include the following:

A. Ensuring airway patency;
B. Supporting adequate ventilation;
C. Identifying and managing significant hemorrhage, which could be abdominal, pelvic, or intracranial.

The application of Advanced Trauma Life Support (ATLS) guidelines to pediatric trauma care significantly impacts survival and long-term outcomes [38,39]. Moreover, less invasive procedures have shown to improve outcomes in pediatric intensive care units [30,40,41,42].

Pediatric trauma care expertise includes daily management of pediatric patients with certified competency training [40,41], which are best carried out in team [30,43]. The composition of the pediatric trauma team and the specialty of the trauma team leader might differ significantly from center to center. More than the role itself, it is paramount that each team works well together, covering every step of the ATLS procedure harmoniously [39,44]. In the present manuscript, the focus is on the initial management of the PPT/PMT from an orthopedic and traumatology point of view and as performed according to the local protocol in our institution, which is an academic level I pediatric trauma center.

## 2. Materials and Methods

This work is based on a retrospective analysis of monocentric prospectively collected data. According to our trauma network, currently all cases of PPT ≤ 12 years old in a 10 million-populated region are centralized to our hospital, whilst those >12 years old are distributed among all 6 trauma centers of our region [45]. Thus, our center is the only trauma center for pediatric populations ≤12 years in the region and for a 1 million population of children >12 years. As a Level 1 trauma center, patients in our institution are treated routinely for polytrauma in adults. However, in the present study, we report only data related to patients <18 years of age. In our trauma network, the severity of the pediatric trauma is assessed according to the Pediatric Trauma Score (PTS) [38,39,40], and a patient is considered a PPT/PMT with a PTS ≤ 8 (Table 1). All patients <18 years old and with a PTS ≤ 8 admitted alive to our institution were enrolled. Patients who died at the accident site or who died during transport to the hospital were excluded. The study was conducted in accordance with the Declaration of Helsinki and approved by the Institutional Review Ethics Committee of Bergamo (ITA), using data from an ongoing register. Patients and/or patients’ parents/guardians (according to age and clinical situation) provided informed consent for the use and sharing of their health information, including images; all data were anonymized and, whenever possible, used as aggregated data. In our center, the pediatric trauma team for the 1st evaluation (according to ATLS) was undertaken by a pediatric anesthesiologist (basically, for A and B of the ATLS protocol); a pediatric surgeon (for B and C); a dedicated nurse; a radiologist; and an on-call orthopedic surgeon and neurosurgeon who remained on stand-by for the 1st evaluation, according to the judgment of the trauma team leader. The need for emergency treatment for musculoskeletal injuries of the extremities is usually associated with massive bleeding (“C”), not due to bone injuries or soft tissue lesions themselves. The first hour following a severe injury is crucial, as prompt and appropriate medical intervention is critical for maximizing a patient’s chances of survival and minimizing long-term complications. Therefore, this first hour is called the “golden hour of shock” [37,44,46,47]. Open fractures require urgent treatment but are not usually priorities managed during the “golden hour of shock” [35,36]. Therefore, during the 1st evaluation, a traumatological evaluation is performed only if required by the trauma team leader, whilst during the 2nd evaluation, every PPT/PMT case receives a traumatological evaluation (a head-to-feet evaluation) [39].

Among the large quantity of data concerning PPT and traumatology, we focused here on the management of the PPT during the golden hour of shock, which is the most critical time period for the outcomes and requires impressive teamwork. Probably more than in other situations during the management of PPT, every specialist must think while knowing the needs and indications of the other caregivers involved. Particularly critical in the golden hour of shock is the decision regarding the type of treatment to be administered following the principles of early appropriate care (EAC), ranging from systemic damage control (DCO) to early total care (ETC) [39,47,48,49].

Due to the difficulty of extracting precise data among an enormous quantity of variables over a long period of time (10 years), we chose to analyze different aspects over three different periods:

- A 10-year period was chosen to analyze the diaphyseal femur fracture. Diaphyseal femur fractures were extracted as indicators of DCO versus ETC, according to the expected frequency in association with PPT and univocal classification in registers. We also chose to observe retrospectively the treatment approach (temporary external fixation or definitive treatment).
- A 4-year period (2021–2024) was chosen to describe the associated lesions of PPT, such as head injuries, thoracic and abdominal lesions, spinal lesions with or without neurological consequences, and major blood vessel lesions.
- A 1-year period was chosen to analyze the overall in-hospital mortality and admission rates in the PICU.

## 3. Results

Over a 10-year period, among the 298 diaphyseal femur fractures treated in our center, 84/298 cases (28%) presented at least one other associated lesion and 46/298 cases (15%) were classified as PPT and underwent damage control (DCO) for femur fracture(s).

DCO was performed according to age as follows:

- In the age-group 15–17 years, 23/23 (100%) cases were treated with temporary external fixation (ExFix);
- In the age group 12–14 years, 9/14 (64%) cases were treated with ExFix, and 5/14 (26%) cases were treated with elastic stable intramedullary nails (ESIN);
- In the age group 5–11 years, 1/5 (20%) cases were treated with ExFix, and 4 cases were treated with ESIN;
- In the age group 0–4 years, 2/4 (50%) cases were treated with ESIN, and 2/4 (50%) cases were treated with a hip spica cast.

The minimum age for DCO of femur fractures with ExFix was 10 years. No patients died during these procedures.

In the 4-year period, 2021–2024, 60% of the PPT cases had a head injury, 30% had least one major musculoskeletal injury (pelvic and/or long bone fracture(s) or (sub)amputation), 25% had thoracic lesion(s), 18% had abdominal lesion(s), 16% had a spinal injury (with or without neurological consequences), and 5% had lesions in a major blood vessel (the aorta or one main trunk). Even considering the fact that the referring population ≤12 years is 10 times bigger than the population >12 years (10 vs. 1 million inhabitants), the frequency of admissions per year for patients ≤12 years was 15% of patients >12.

In 2019, 193 patients <18 years old were admitted to the emergency room as PPT/PMT cases; 115 were ≤12 years old; and 78 were > 12 years old. Initially, 46% were admitted to the PICU, and 32% needed a period ≥24 h in the PICU; finally, 65% were admitted to traumatology (including patients initially cared for in the PICU). The overall in-hospital mortality rate was 7%.

## 4. Discussion

According to present data, the in-hospital mortality of PPT was 7%, which is a similar rate described by other authors [3,7]. PPT/PMT is a complex pathology that needs a multidisciplinary approach in dedicated centers [21,35,50,51]. The management starts long before the arrival in hospital at the accident site: a trauma network is paramount to triage, centralize, and start the resuscitation and first treatment [30,32,37,42,46,52,53,54]. For these reasons, the definition itself of PPT/PMT is a critical issue, influencing the first steps of management (triage, resuscitation, and centralization) and the registers and epidemiology provided for PPT cases, which is necessary to create and maintain the trauma network in different healthcare systems and measure the necessary costs and resources [5,17,29,31,34,35]. In particular, the definition of “pediatric” is not established or clear-cut between children, adolescents, and adults. Adolescents are mostly treated like adults, whilst children <5 years seem to have a higher intra-hospital mortality rate in comparison with older patients, and this could indicate the most critical age group in terms of management [26,29,50].

For clear safety reasons, there is a tendency to over-triage the patients and overrate the real number of PPT/PMT cases [29,55,56,57]. According to a disaster plan for mass casualty incidents involving the pediatric population in a metropolitan area, to provide the most effective and efficient response, patients can be divided into age groups as follows: ≤3 years old, 3 < years ≤ 12, and >12 years. This arbitrary classification considers the resuscitation skills and hospital capabilities of a general hospital to handle pediatric patients. Centers in which both pediatric and adult trauma are treated could have fewer concerns in this regard, considering transitional age groups [29].

The focus of the present work is on the traumatological point of view; therefore, for a systemic approach to PPT/PMT, we refer to appropriate readings [37]. The (pediatric) orthopedic surgeon usually starts working after Airways and Breathing (“A” and “B” of ATLS) have been addressed. ATLS has been developed to function with a single health care professional; however, working in a team, evident bleeding sources are stopped with compression, and grossly misaligned limbs are splinted simultaneously to A and B if this does not disturb the workflow. The schema followed to treat major trauma according to the ABCDE schema has been updated as proposed by the ATLS [37,47], and “stop the bleed” has become the first step XABCDE (“X” is for massive open bleeding). This sequence was already embedded in the tactical emergency casualty care (TECC), but not used among civilian care givers in hostile environments, which uses an algorithm called MARCH (M—Massive bleeding, A—Airway Management, R—Respiration, C—Circulation, and H—Head injury/Hypothermia/Hypovolemia) [2,58,59,60].

In PPT/PMT, the techniques of reduction and fixation are basically the same as those used for isolated injuries [44]. During the “golden hour of shock”, the role of the orthopedic surgeon in managing the physiology of PPT/PMT is committed to hemodynamic stability and stopping any bleeding: hypovolemic shock associated with pelvic injuries, (massive) limb hemorrhages, and multiple long bone fractures can be considered emergencies. An isolated femur fracture in children associated with hemodynamic instability (or otherwise explained blood loss) is an imperative indication to look for other associated injuries [61].

After the first evaluation, the main orthopedic issues during the initial management of a PPT/PMT can be reassumed with the following questions, which will be discussed separately in the following subchapters:

1. Is emergency treatment needed? When should the fracture be fixed?
2. Is a temporary damage control surgery (DCO) indicated?
3. Does the PPT/PMT-patient need a different treatment method because of his/her general conditions?

### 4.1. Is an Emergency Fixation Needed? When Should the Fracture Be Fixed?

**Figure 1:**
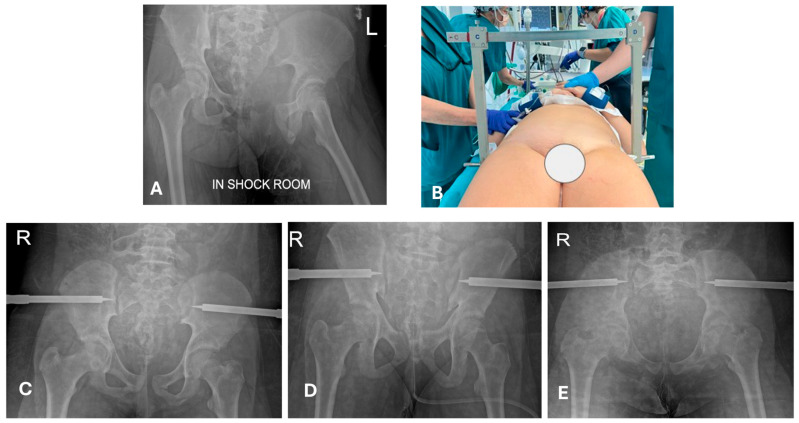
A 12-year-old girl, after high-energy trauma (motocross accident: direct frontal collision with a tree during a jump). (**A**) Unstable open pelvic ring fracture. The patient was hemodynamically unstable upon admission. The exposure of the fracture was in a cavity (the left pelvic ramus protruded through the vagina), which is a lesion that might be easily missed during the primary evaluation without a systematic approach and exposure of the patient. (**B**) Pelvic fixation in an emergency with a pelvic C-clamp. (**C**–**E**) Postoperative radiographs (antero-posterior and outlet and inlet views, respectively). The patient survived and followed definitive treatment in the following days. Courtesy of Dr Garcia Parra C. and Pelis A (Papa Giovanni XXIII Hospital, Bergamo), with permission.

In a pediatric patient, organ failure usually develops soon after injury and tends to resolve quickly after skilled resuscitation and management [62,63]. Therefore, if the need for osteosynthesis is urgent, it can be undertaken in an early stage of multisystem compromise [21]. The difference between early total care (ETC) and DCO is vaguer in comparison to adults [23,61]. Definitive pediatric fixation techniques are usually less invasive in comparison to adult ones; hence, a second hit due to surgery is a less determinant variable in the treatment choice [49,64,65]. Even when considering the surgical time needed to fix a fracture with elastic stable intramedullary nails (ESINs) in comparison to external fixation, the differences do not seem clinically relevant [66]. Essentially, reduction and fixation in an emergency are needed to treat bleeding (for example, pelvic ring fractures in hemodynamically unstable patients or bone fragments tethering a vessel); limb-threatening lesions can be considered an emergency (for example, those that require sub-amputation) [47,64] (Figure 1).

Particularly in PPT, the sum of more lesions must be carefully considered, and one single fracture might contribute substantially to a hemorrhagic shock (e.g., a combination of abdominal and long bone fracture). The strategy starts from the expected major source of bleeding (e.g., the trunk), immediately followed by the stabilization of the fracture (e.g., the femur), as simultaneous treatment is not always practicable. Multiple long bone fractures at the same time might maintain a hemorrhagic shock by itself; therefore, emergency fracture stabilization is necessary. A stabilization in *emergency* basically means that it has to be carried out as soon as it is recognized, before further diagnostics are performed (exactly as the ABCDE process describes during primary evaluations in ATLS [46]), but this is rare in children. An example of stabilization carried out in an emergency is a pelvic ring fracture associated with hemodynamic instability. In these situations, other fractures and dislocations are urgencies (not emergencies) and can be managed out of the golden hour and the secondary evaluation with a completed diagnostic [59,60].

### 4.2. Is a Temporary Damage Control Surgery (DCO) Indicated?

**Figure 2:**
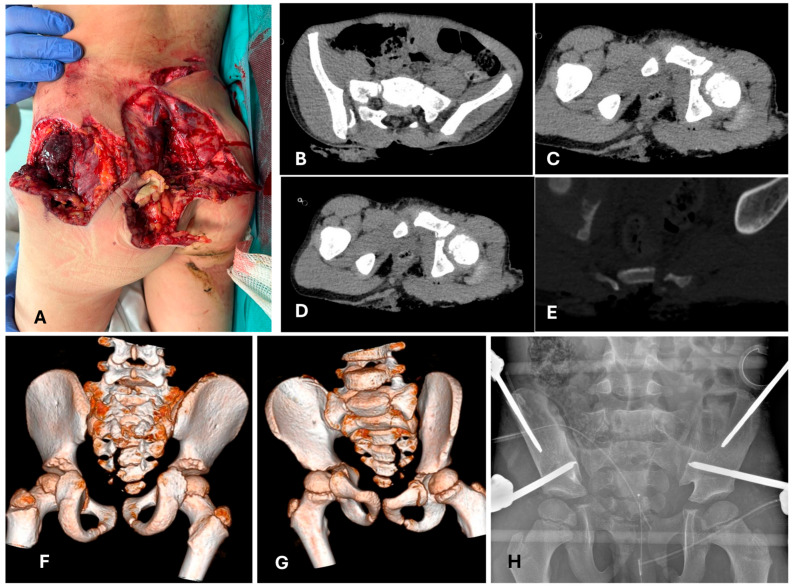
A 7-year-old patient, pediatric major trauma (PMT), accidentally run over by a snowcat. (**A**) Clinical presentation of the open sacral and pelvic fractures (back-view). (**B**–**E**) Computed tomography at admission; triplanar and (**F**,**G**) 3D reconstruction. Note the avulsion of the Risser from the right side, which corresponds to the closed book side of the pelvis, and the open sacral fracture, as described by Oransky et al. [25]. (**H**) Systemic (and local) damage control with temporary external fixation of the pelvis. A supra-acetabular and iliac configuration has been chosen according to both the instability of the patient and the local soft tissue situation.

**Figure 3:**
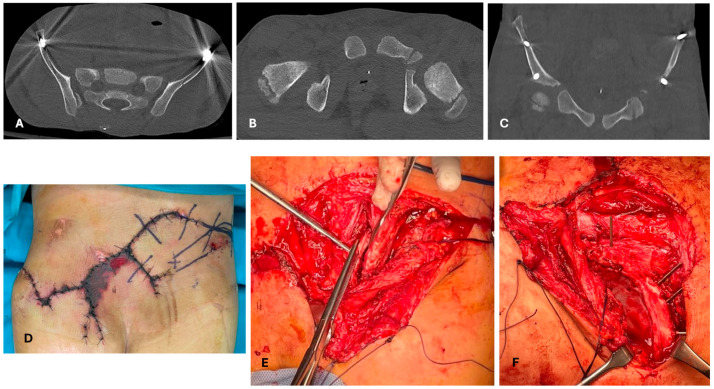
The same patient as Figure 1. CT check for planning, with ExFix in situ. (**A**–**C**) Triplanar reconstruction. (**D**) The image shows the situation at 5 days post-trauma and the time of definitive treatment. (**E**) Intraoperative findings: the Risser is completely detached from the iliac wing, starting from the posterior-superior iliac spine to the anterior third of the iliac wing. (**F**) Intraoperative finding after reduction in the iliac wing and Risser; fixation is performed with K-wires [25].

**Figure 4:**
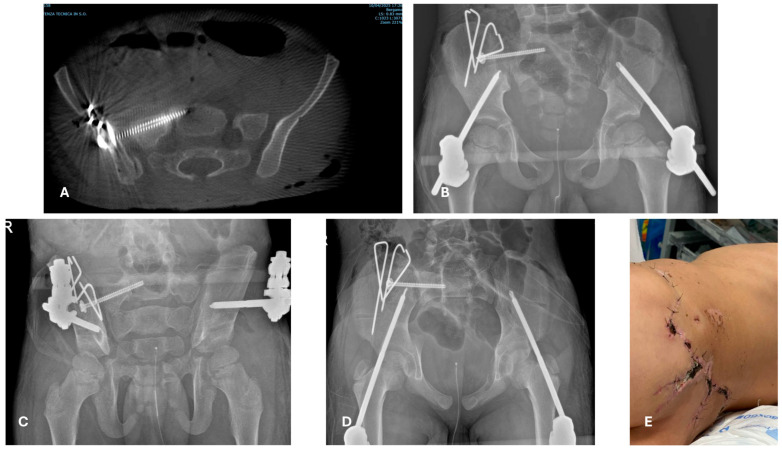
The same patients as Figure 1 and Figure 2. Postoperative controls. (**A**) Position of the sacro-iliac screw, checked with an O-arm. (**B**) Antero-posterior, (**C**) outlet, and (**D**) inlet views of the pelvis. (**E**) Skin situation at around 10 days after the operation.

**Figure 5:**
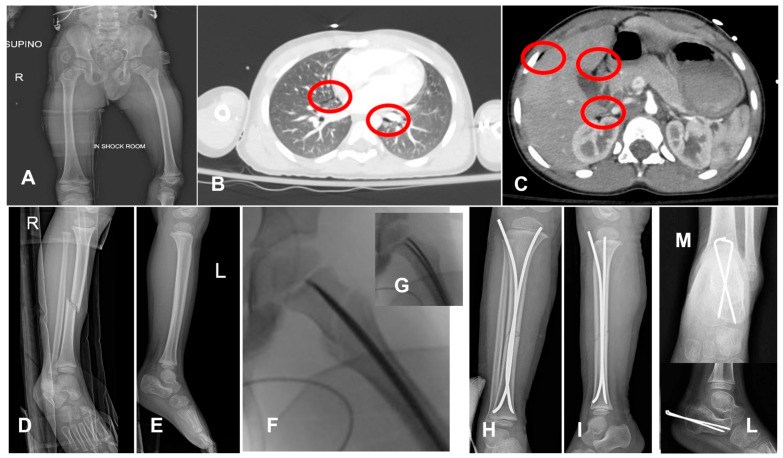
A 4-year-old boy who fell from a balcony. After the 1st and 2nd evaluation, the patient was identified as hemodynamically stable, with concussion, minimal pneumomediastinum, (red circles, in (**B**)) pneumoperitoneum (red circles, in (**C**)), a femur neck fracture, (**A**) right-leg diaphyseal fracture (**D**), and left calcaneus fracture (**E**). The chosen strategy was DCO and observation of the abdominal and thoracic lesions. Closed reduction and internal fixation with ESIN of the proximal femur (**F**,**G**), tibia (**H**,**I**), and with Kirschner wires of the calcaneum (**L**,**M**). Calcaneus would have been treated conservatively in other cases, and this choice might be discussed; however, management in the pediatric intensive care unit is easier without a cast/brace.

Early appropriate care (EAC), also called safe definitive surgery (SDS), is a concept developed to merge early total care (ETC) and damage control in orthopedics (DCO) [48,67]. Systemic DCO uses fast and less invasive procedures to avoid a second hit due to additional surgical stress [49,65,67,68]; in traumatology, this is mostly intended as temporary external fixation. In EAC/SDS, definitive treatment is carried out with respect to the momentary evolution of clinical conditions, so that definitive treatment can be flexibly shifted to DCO and vice versa. Concerning pelvic injuries, in our experience, their management does not substantially deviate from that of adults (Figure 1 and Figure 2), but it must be underlined that these are rare lesions in children. Here, “evidence-based” information is more *experience-based* than *data-based*. In children, the indication for DCO is less supported by evidence; in addition, definitive fixation devices are usually less invasive in comparison to adults [21,69] (Figure 2, Figure 3 and Figure 4). For example, diaphyseal long bone fractures can be treated with ESIN or conservatively, instead of using reamed nails; therefore, a second hit (regarding debris, blood loss, and surgical time) caused by ESIN or conservative treatment is expected to be similar to temporary external fixation [66]. Looking at our data relative to diaphyseal femur fractures, out of 298 consecutive pediatric diaphyseal femur fractures, 84/298 (28%) presented with at least one other associated lesion, and 45/298 (15%) were classified as PPT/PMT (Figure 5) [26]. DCO is a procedure performed in cases of major trauma and even in children, but it might not be a form of temporary external fixation in the pediatric population. In other words, DCO is a timeframe during EAC, not an implant: DCO can be achieved through different treatment methods (e.g., ExFix, ESIN, or braces).

### 4.3. Does the PPT/PMT Patient Need a Different Treatment Method Because of His/Her General Conditions?

**Figure 6:**
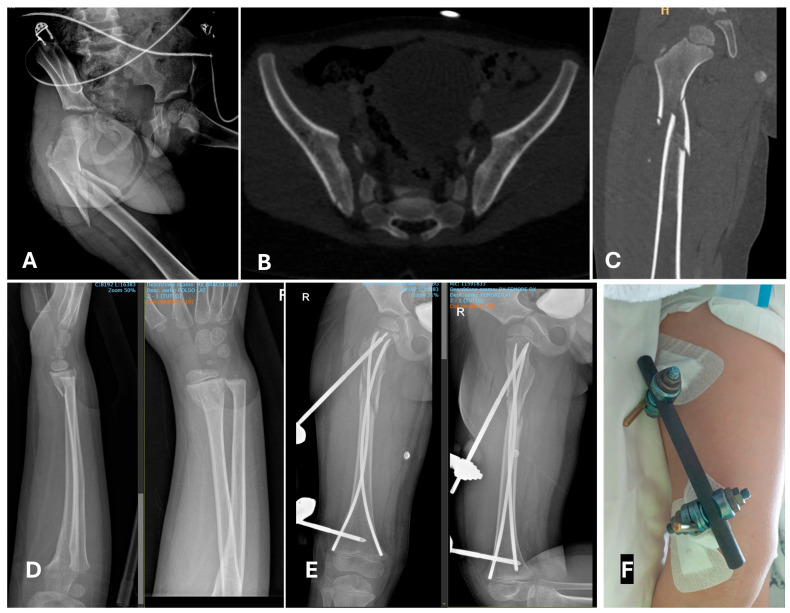
A 4-year-old boy who fell with a bicycle down some stairs. After 1st and 2nd evaluations, a proximal diaphyseal closed multifragmentary fracture was identified (**A**,**C**), no dislocated pelvic fracture (**B**), and a closed forearm fracture (**D**). The chosen approach was early total care: ESIN and an external fixator “missing the nail” for the femur (**E**,**F**) and a cast for the forearm.

In some cases, a musculoskeletal lesion in PPT/PMT might be treated differently from the same lesion as an isolated injury. Fractures that are usually treated conservatively (e.g., femur shaft or humeral shaft fractures) have a low threshold for surgical fixation [69]. In the authors’ experience, this occurs mostly due to appropriate management of a multidisciplinary and complex patient, who needs more invasive procedures with different contamination settings and careful mobilization in the PICU; for the same reasons, when both internal and external fixation devices are possible options, the internal one might be preferred. However, in contrast to adults, the choice of a more aggressive approach to the fractures to achieve fast weight-bearing in critical children and babies is usually not an issue (Figure 5F–I and Figure 6E). Fractures in babies are almost always treated conservatively with casts and splints adapted for intensive monitoring or concomitant abdominal procedures. The use of circular/spica casts must be carefully applied in PPT/PMT during the first days: rapid body volume changes in NICU/PICU are rare. The identification of the source of pain might be particularly challenging with the patient under general anesthesia, and the diagnosis of compartment syndrome might be delayed or overseen. For these reasons, when both surgical and conservative treatment are an option, the indication could shift to surgery.

As mentioned above, when deciding between DCO versus definitive treatment in PPT/PMT cases, different options might be considered. Temporary external fixation is an option, but might not always be the best one [70,71,72,73,74]. The use of minimally invasive techniques to stabilize fractures results in minimal blood loss and reaming debris [49,65,69], and even the surgical time might not play a role [66]. In other words, DCO in children can coincide with definitive treatment; external fixation, ESIN, and possibly minimally invasive plate osteosynthesis (MIPO) might all be considered as options. For complex fracture patterns, a useful option to consider is a combination of ESIN and external fixation (the miss-a-nail technique) [75] (Figure 6).

Another situation to consider when contemplating different treatment methods is mass casualties. In our center, we have not experienced this kind of situation and refer to the experiences of other authors. When the trauma network is overloaded, a (temporary) external fixation could be considered, as these types of treatment are easily stocked in large quantities and can be sterilized and reused for nearly all kinds of fractures. Patients might then be back-transported to other centers or wait for definitive treatment [76,77]. Accordingly, in each center, a reasonable stock of ready-to-use devices should be available.

In the rare case that one or more fracture(s) need(s) to be stabilized because of hemodynamic instability, a fixation is in any case needed (DCO through external fixation, ESIN, or even a spica cast).

The present study has some limitations. Firstly, only data regarding the overall in-hospital survival rate and treatment methods used for the diaphyseal femur fracture were reported, and even if these variables might be used as markers, they are not the outcome. Data were reviewed retrospectively in a single institution. More healthcare givers were involved in the treatment during the period observed; therefore, according to the authors, the results are not generalizable. Although the ATLS is a well-established method, the variables are countless and involve both the clinical situation of each single patient and the application of the ATLS method in every single center. PPT/PMT is a rare disease, particularly for children <5 years old [78]: the number of cases in the present study is valuable, in the authors’ opinion, to define this work as a starting point for further investigations. However, not enough cases are included for solid statistical inference and evidence-based conclusions.

## 5. Conclusions

PPT/PMT is a rare condition with better results when managed in multidisciplinary dedicated teams in dedicated centers. In our institution, pediatric trauma is assessed with the Pediatric Trauma Score, and the workup follows the ATLS guidelines, implemented by a dedicated trauma team. The role of the orthopedic surgeon during the primary evaluation of PPT/PMT is to contribute to stopping the bleeding and hemorrhagic shock. The need for emergency treatment of musculoskeletal injuries is rare and is usually associated with massive bleeding, not based on bone and soft tissue lesions themselves. Open fractures need urgent treatment, but these are not usually priorities treated during the “golden hour of shock”. In PPT/PMT cases, the DCO in adolescents is superimposable to adults, whilst in babies and children, DCO is still performed, but it is not a form of temporary external fixation.

## Figures and Tables

**Table 1:** Pediatric Trauma Score. The minimal score is −6 and the maximum score is +12. Mortality is estimated at 9% with a PTS > 8, and at 100% with a PTS ≤ 0. There is a linear relationship between the decrease in PTS and the mortality risk (i.e., the lower the PTS, the higher the mortality risk). [38,40].

Pediatric Trauma Score (PTS)	+2	+1	−1
**Weight**	>20 kg	10–20 kg	<10 kg
**Airway**	Patent	Maintainable	Unmaintainable
**Systolic blood pressure**	>90 mmHg	50–90 mmHg	<50 mmHg
**Central nervous system**	Awake	Obtunded/Loss of consciousness	Unresponsive
**Fractures**	None	Closed or suspected	Multiple (closed or open)
**Wounds**	None	Minor	Major, penetrating or burns

## Data Availability

The data presented in this study are available in anonymous form from the corresponding author on request due to privacy issues.

## Abbreviations

The following abbreviations are used in this manuscript:

ETC	Early Total Care
PPT	Pediatric Polytrauma
PMT	Pediatric Major Trauma
DCO	Damage Control in Orthopedics
ESIN	Elastic Stable Intramedullary Nail(s)
ExFix	External fixation
NICU	Neonatal Intensive Care Unit
PICU	Pediatric Intensive Care Unit
SDS	Safe Definitive Surgery
